# Atmospheric oxidation of dimethylsiloxanes, a source of Si=O double bonds?

**DOI:** 10.1007/s11356-025-37108-6

**Published:** 2025-11-14

**Authors:** Christoph Rücker, Dennis Troegel, Klaus Kümmerer

**Affiliations:** 1 Institute for Sustainable Chemistry, Leuphana University Lüneburg, Universitätsallee 1, 21335 Lüneburg, Germany; 2 Department of Applied Chemistry, Nuremberg Institute of Technology, Kesslerplatz 12, 90489 Nuremberg, Germany

**Keywords:** Oligosiloxanes, Hydroxyl radical, Environmental degradation, Atmospheric pollution, Atmospheric siloxanes, Silanones, Bicyclic siloxanes, MOLGEN 5.0

## Abstract

Volatile methylsiloxanes are pollutants in the atmosphere and are oxidized therein by hydroxyl radicals to siloxanols and other product classes. Under (simulated) atmospheric conditions, some stable oxidation products were recently obtained that were assigned by their authors to structures comprising Si=O double bonds, based on molecular formulas only that were derived from mass spectrometry. Here we compare the stability and even isolability of such compounds to what is known about Si=O double bonds and the few known compounds comprising them. The latter are highly reactive, thermodynamically and kinetically labile, and cannot be isolated except when stabilized by strong steric or electronic substituent effects. Si=O comprising compounds without such effects are at most observable at a very low temperature (12 K) in an argon matrix. In siloxane atmospheric oxidation products that are completely stable at ambient temperature and above, no stabilizing substituents are present. Therefore, structures featuring Si=O double bonds for these products are extremely unlikely. As for alternatives, we consider structures that instead of a Si=O double bond contain an additional ring, e.g., a Si–O–Si bridge, and a possible mode of such bridge formation. Our proposed structures, in contrast to those with Si=O double bonds, are compatible with all experience; in particular, they explain the stability of these compounds.

**Graphical abstract:**

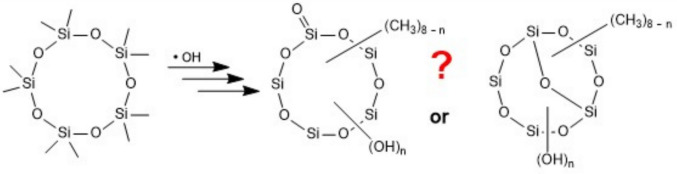

**Supplementary information:**

The online version contains supplementary material available at 10.1007/s11356-025-37108-6.

## Introduction

Dimethylsiloxanes (siloxanes for short) are anthropogenic chemicals comprised of –Si(CH_3_)_2_–O– units in a linear, branched, or cyclic arrangement. They are produced worldwide in several million tons per year and are used in many industrial sectors and by consumers (Rücker and Kümmerer 2015). For structures, nomenclature, and shorthand designations of siloxanes (M = Me_3_SiO_1/2_, D = Me_2_SiO_2/2_, T = MeSiO_3/2_, Q = SiO_4/2_, Me = CH_3_), see Brook 2000; Rücker and Kümmerer 2015; Rücker et al. 2023a. Cyclic oligomers octamethylcyclotetrasiloxane (D_4_) and decamethylcyclopentasiloxane (D_5_) are high production volume (HPV) chemicals; they are formed as byproducts on an industrial scale in the hydrolysis and condensation of Me_2_SiCl_2_ for the production of polydimethylsiloxanes (PDMS). To a lesser extent, they are synthesized specifically as starting materials for the production of defined silicone oils. D_4_ and D_5_ are also formed by hydrolysis/cyclocondensation or thermal treatment of PDMS. PDMS and/or D_4_ and D_5_ are contained in many industrial and consumer products (Moretto et al. 2000; Horii and Kannan 2020). D_4_ and D_5_ are PBT (persistent, bioaccumulative, and toxic) and vPvB (very persistent and very bioaccumulative) according to the European REACH classification. As such, they are substances of very high concern (SVHC); their use is being regulated (EU 2024), and they are under consideration as persistent organic pollutants (POPs) for inclusion in Annex B of the Stockholm convention (ECHA 2023).

Unless bearing hydrophilic substituents such as OH groups, siloxanes are hydrophobic and poorly soluble in water. Hydrolysis of most siloxanes (Si–O bond cleavage) in the aqueous phase via siloxanol intermediates to finally dimethylsilanediol (Me_2_Si(OH)_2_, DMSD) is slow at near-neutral pH but can be catalyzed by both acid and base (Ducom et al. 2013; Rücker et al. 2023b). The Si–C bond likewise is rather robust, inert to usual ambient reagents such as water, oxygen, or visible/UV light, and biodegradation of siloxanes in the sense of microbial Si–C bond cleavage in the environment has not been demonstrated (Rücker et al. 2023b), though recently a cytochrome P450 enzyme variant was obtained by directed evolution that cleaves a Si–C bond in small linear dimethylsiloxanes (MM, MDM) or D_4_ (Sarai et al. 2024). Lower dimethylsiloxanes such as D_4_ and D_5_ have rather high vapor pressures, and their emissions from consumer use are mostly to the air. Emissions to water also largely volatilize into the atmosphere (Gallego et al. 2020; McLachlan 2020), where they are found both in the gas phase and in aerosol particles (Bzdek et al. 2014; Kim and Xu 2016; Gallego et al. 2020; Katz et al. 2021; Brown et al. 2021; Cheng et al. 2021; Chen et al. 2023; Cao et al. 2023; Wania et al. 2023; Kang et al. 2023; Zhang et al. 2024). Even siloxanes of higher molecular mass and correspondingly lower volatility were recently detected in aerosol particles in diesel engine exhaust (Yao et al. 2022, 2023), probably originating from siloxane lubrication oils or siloxane-based defoaming agents (Ren et al. 2023; Perez et al. 2024).

Nonamethylcyclopentasiloxanol (D_4_D^OH^, also known as D_4_TOH), a semivolatile oxidation product of D_5_, was detected both in particulate matter (PM_2.5_) and in the gas phase in ambient air in two U.S. cities, as the first oxidized dimethylsiloxane derivative found in outdoor air (Milani et al. 2021). D_4_D^OH^, D_3_D^OH^_2_, and isomers of both were recently found in New York City aerosol (Meepage et al. 2024). Dimethylsilanediol, the final hydrolysis product of PDMS and D_*n*_, volatilizes from soil or through plants into the air (Xu et al. 2024a, b). Consequently, environmental mineralization of siloxanes to CO_2_, H_2_O, and SiO_2_ will occur mainly in the atmosphere, initiated by reaction with OH radicals or Cl atoms. It is therefore important to understand the atmospheric chemistry of siloxanes, including at least to know the products of initial oxidation steps. In recent years, several authors reported finding, among the products of laboratory experiments on atmospheric siloxane oxidation, some particular oxidized dimethylsiloxanes whose structures were envisaged to comprise a Si=O double bond (Wu and Johnston 2016, 2017; Divsalar et al. 2018; Avery et al. 2023; Chen et al. 2023; Meepage et al. 2024). Since Si=O double bonds are extremely unusual in chemistry, we in the present work try to shed some light on the identity of these oxidized siloxanes and provide alternative structures for the observed molecular formulas. We first give an overview of known (conventional) siloxane oxidation products, then focus on the new products that purportedly contain or may contain Si=O double bonds. We consider the reactivity of known Si=O compounds to be compared to that of the compounds in question. We recall that, stoichiometrically, a double bond is equivalent to a cycle, and based thereon suggest possible alternative structures for particular compounds found by the above authors.

## Products of gas phase reactions of siloxanes with OH radicals under simulated environmental conditions

### Conventional products

While reactions of siloxanes with OH radicals were occasionally observed in the aqueous phase (Buch et al. 1984; Xu et al. 2017; Han et al. 2020; Tang et al. 2020; Saito et al. 2020; Kim et al. 2024) or on solid surfaces (Sun et al. 2003; Lamaa et al. 2014), insight into product identity was obtained primarily from laboratory experiments in the gas phase (smog chambers or oxidative flow reactors) with various methods of OH radical generation and under various conditions (humidity, temperature, concentrations, exposure, etc.). Table 1 lists the particular products and compound classes reported from individual siloxanes. Often silanols were found as major products (*Si*–CH_3_ → *Si*–OH, Sommerlade et al. 1993; Atkinson et al. 1995; Markgraf and Wells 1997; Tuazon et al. 2000; Chandramouli and Kamens 2001; Safron et al. 2015; Wu and Johnston 2016, 2017; Alton and Browne 2020, 2022; Kang et al. 2023; Meepage et al. 2024; Lewine et al. 2025). *Si* (in italics) here means a Si atom plus its three substituents not involved in a reaction; the arrow indicates replacement of a methyl group by a different substituent. Formyloxysilanes (silyl formates) were also detected (*Si*–CH_3_ → *Si*–OCHO) and may or may not be the immediate precursors of silanols by hydrolysis under the reaction conditions (Atkinson et al. 1995; Alton and Browne 2020, 2022; Lewine et al. 2025). Even a hydroperoxymethoxysilane was reported (*Si*–CH_3_ → *Si*–OCH_2_OOH, Alton and Browne 2022). These observations demonstrated Si–C bond cleavage being an important degradation result in the treatment of siloxanes with OH radicals in the gas phase. Along with products of Si–C bond cleavage, various products with an intact Si–C bond were reported, such as hydroperoxymethylsilanes (*Si*–CH_3_ → *Si*–CH_2_OOH, Sommerlade et al. 1993; Xiao et al. 2015; Alton and Browne 2022), hydroxymethylsilanes (*Si*–CH_3_ → *Si*–CH_2_OH, Sommerlade et al. 1993; Xiao et al. 2015; Wu and Johnston 2016), and a formylsilane (*Si*–CH_3_ → *Si*–CHO, Xiao et al. 2015). Moreover, “dimers” were found in which two cyclic siloxane moieties are connected by an ethylene fragment (2 *Si*–CH_3_ → *Si*–CH_2_CH_2_–*Si*), by an oxygen atom (2 *Si*–CH_3_ → *Si*–O–*Si*, Sommerlade et al. 1993; Wu and Johnston 2016, 2017; Lewine et al. 2025), or by a methylene group (2 *Si*–CH_3_ → *Si*–CH_2_–*Si*, Wu and Johnston 2016, 2017). Dimers linked by CH_2_ as well as product Me_3_SiCH_2_OH obtained from D_5_ (Wu and Johnston 2016) exhibit newly formed Si–C bonds. Hydrolysis (Si–O bond cleavage) reactions also played some role, as demonstrated by the formation of products containing fewer Si atoms than the starting materials, such as Me_3_SiOH from both MM and MDM (Markgraf and Wells 1997). Conversely, products containing more (but not twice as many) Si atoms than the starting materials, such as D_4_ from MDM, and D_3_ formation from MDM, suggested condensation and cyclocondensation (formation of new Si–O bonds, *Si*–OH + HO–*Si* → *Si*–O–*Si* + H_2_O) (Markgraf and Wells 1997; Tuazon et al. 2000). During these and other studies, it became obvious that the oxidation products are less volatile and less hydrophobic than the parent compounds and will partition between gas and aerosol phases in the atmosphere (Chandramouli and Kamens 2001; Janechek et al. 2019; Shah et al. 2020; Charan et al. 2022; Xu et al. 2022; Han et al. 2022; Avery et al. 2023; Chen et al. 2023; Zhang et al. 2024; Lewine et al. 2025).

**Table 1:** Conventional products reported from reaction of siloxanes with hydroxyl radicals in the gas phase

Starting material	Products^a^	Reference
D_4_	D_3_D^OH b^, D_3_D^CH2OOH^, D_3_D^CH2OH^, D_3_D^CH2CH2^DD_3_, D_3_D^O^DD_3_^b^	Sommerlade et al. 1993
MM (L_2_)	MM^OCHO^, MM^OH b^, HCOOH	Atkinson et al. 1995
Me_4_Si	Me_3_SiOCHO	
MM	Me_3_SiOH, MM^OH^	Markgraf and Wells 1997
MDM (L_3_)	MDM^OH^, MD^OH^M, Me_3_SiOH, MM^OH^, D_3_, D_4_	
MD_2_M (L_4_)	Many minor products	
Me_2_Si(OH)_2_	MeSi(OH)_3_, HO(SiMe_2_O)_2_H, HO(SiMe_2_O)_3_H, D_3_	Tuazon et al. 2000
Me_3_SiOH	Me_2_Si(OH)_2_, MeSi(OH)_3_	
D_5_	D_4_D^OH b^	Chandramouli and Kamens 2001
D_4_	D_3_D^OH^	Safron et al. 2015
D_5_	D_4_D^OH^	
D_6_	D_5_D^OH^	
D_3_	D_2_D^CH2OH^, D_2_D^CH2OOH^, D_2_D^CHO^	Xiao et al. 2015
D_5_	D_4_D^OH^, D_4_D^CH2OH^, D_4_D^CH2^DD_4_, Me_3_SiCH_2_OH, Si_<5_, Si_6–9_ products Si_10_ products	Wu and Johnston 2016, 2017
D_4_	Products oxidized at multiple positions	
	Products of similar types	
D_4_	D_3_D^OH^, D_3_D^OCHO^, D_2_D^OH^_2_, D_2_D^OCHO^_2_, D_2_D^OH^D^OCHO^	Alton and Browne 2020
D_3_	As above and D_2_D^OH^, DD^OH^_2_, D_2_D^OCHO^, DD^OH^D^OCHO^, D_2_D^OCH2OOH^	Alton and Browne 2022
D_4_	As above	
D_5_	D_4_D^OH^, D_3_D^OH^_2_, D_4_D^OCHO^, D_3_D^OH^D^OCHO^, D_4_D^OCH2OOH^, D_4_D^OOH^	
D_5_	D_4_D^OH^, D_3_D^OH^_2_, D_4_D^OCHO^, D_3_D^OH^D^OCHO^, HCHO, HCOOH	Kang et al. 2023
D_5_	Products of multiple Si–CH_3_ → Si–OH replacements	Avery et al. 2023
	Products of type formylsilane and silyl formate, Si_10_ products	
D_5_	D_*n*_D^OH^ and D_*n*_D^OH^_2_ (*n* = 1–3)	Chen et al. 2023
	Products of multiple Si–CH_3_ → Si–OH replacements	
	Products containing both D^OH^ and D^CH2OH^ units	
D_5_	Products of multiple Si–CH_3_ → Si–OH replacements	Meepage et al. 2024
	D_4_D^OH^ and an isomer, several isomeric D_3_D^OH^_2_	
D_5_	D_4_D^OH^, products of multiple Si–CH_3_ → Si–OH replacements	Lewine et al. 2025
	D_4_D^OCHO^, Si_10_ products	

^a^In the siloxane shorthand, a superscript denotes a group that replaces a methyl group, e.g., D_3_D^OH^ is the cyclotetrasiloxane bearing seven methyl and one hydroxyl groups

^b^The structure of this product was verified by comparison with an authentic sample

Reaction at two methyl groups in a single D_4_ molecule, resulting in di-silanol, di-silyl formate and silanol-silyl formate derivatives of D_4_, was observed by Alton and Browne (2020). In 2022, these authors also tracked the appearance of products in time, and from silyl formate and silanol being formed simultaneously rather than sequentially, they concluded that not all of the silanol is formed via silyl formate. In experiments with D_3_, D_4_, or D_5_, these authors did not detect any products with a higher or lower number of Si atoms per molecule than in the starting materials, nor did they report any “unsaturated” (see below) products. From D_3_ and D_5_ also they observed products bearing more than one modified or replaced Me group in the same molecule, and they discussed various theories on reaction paths to the more often observed product types (Alton and Browne 2022; Alton et al. 2023). Oxidation of D_5_ to doubly functionalized derivatives was likewise found by Kang et al. (2023); even multiple replacements of CH_3_ by OH in a single D_5_ molecule were reported (three to six, Avery et al. (2023); up to four, Chen et al. (2023) and Meepage et al. (2024)).

### Products purportedly containing or envisaged to contain Si=O double bonds

**Fig. 1:**
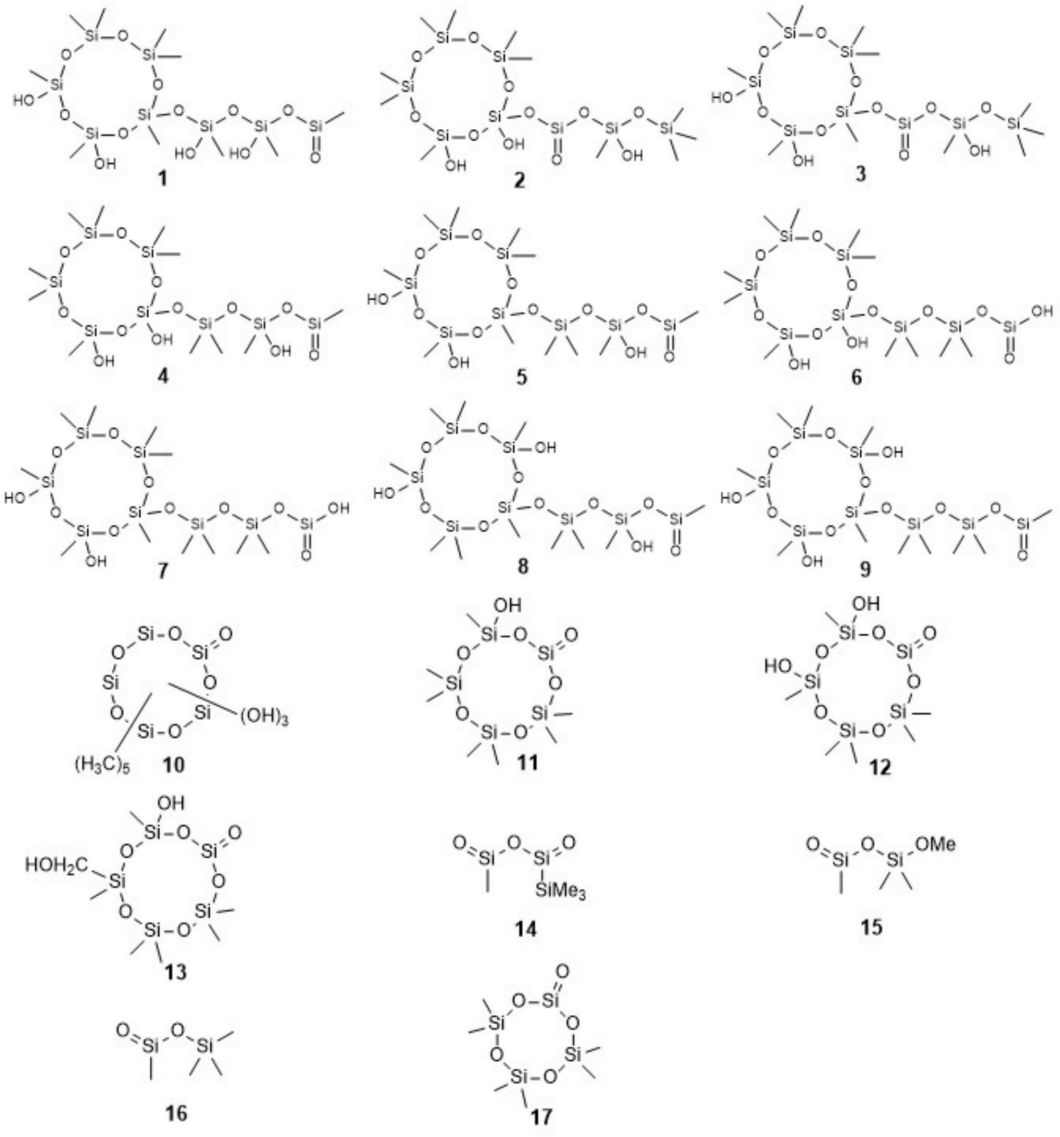
Structures containing Si=O double bonds that were proposed or envisaged in recent literature

The rapid development of mass spectrometry (hardware and software) during the 2010 s enabled scientists to observe in a single experiment many reaction products occurring in a simulated atmosphere simultaneously. Thus, Wu and Johnston (2016) obtained from D_5_ and OH radicals no fewer than 135 unique product molecular formulas of 5, 10, < 5, and 6–9 Si atoms per molecule, the latter two types demonstrating Si–O cleavage, probably by hydrolysis. Among the Si_5_ or Si_10_ products, some had a H/C ratio < 3; these were dubbed “unsaturated”, “indicating the presence of unsaturated functional group(s).” No structures were proposed for these or for similarly defined unsaturated products obtained from D_4_ (out of 529 unique molecular formulas of products, Wu and Johnston 2016). However, in a follow-up paper, the same authors presented candidate structures containing a Si=O double bond for some of the observed stable oxidation products of D_5_ that had Si numbers other than 5 or 10. These structure proposals were based on results of various mass spectrometric techniques, in particular on low hydrogen numbers in molecular formulas. In detail, for a C_10_H_34_O_13_Si_8_ compound they drew candidate structure **1** (Fig. 1), and for a C_11_H_36_O_12_Si_8_ peak they presented eight candidate structures **2**–**9**. The products for which these structures were envisaged were formed at 27 °C and found in aerosol particles that were collected on a glass fiber filter; products were then washed from the filter into a CH_3_CN/H_2_O mixture under sonication for 3 h before MS analysis. No candidate structures without Si=O double bonds were presented (Wu and Johnston 2017). In the same paper, the authors published a list of ~ 200 molecular formulas of products from D_5_, most of which exhibit a more or less strong deficit of hydrogen atoms. No structures were given for these.

Since mass spectrometry, in most applications, is not able to reveal the positions of functional groups within a molecule, these details in structures **1–9** (and **11**–**13**, Fig. 1) are somewhat arbitrary. To illustrate this, we generated and counted all possible positional isomers of **1–9** using MOLGEN-COMB, a software that, given one or several starting materials and one or several generic reaction schemes, generates all possible products up to a predefined number of reaction steps (Kerber et al. 2007). As a result, structure **1** is one out of 575 positional isomeric structures, each made of a cyclopentasiloxane bearing a linear trisiloxane side chain and one Si=O and four Si–OH functions. Similarly, structures **2–9** are just eight out of 286 possibilities to distribute one Si=O and three Si–OH functions over this same siloxane backbone, in both cases neglecting stereoisomerism.

Avery et al. (2023) in experiments with D_5_ and OH radicals or Cl atoms obtained, in both cases, mass spectra containing signals for which they hypothesized structures containing a Si=O double bond. For example, the most intense signal in a mass spectrum of aerosol from D_5_ and OH radicals was assigned the molecular formula C_5_H_18_O_9_Si_5_ and interpreted as the result of three *Si*–CH_3_ → *Si*–OH plus one (unprecedented) Si(CH_3_)_2_ → Si=O steps, i.e., structure **10**. Another high-intensity MS signal was assigned the formula C_4_H_16_O_10_Si_5_ and interpreted as a cyclopentasiloxane structure bearing a Si=O and four OH functions. Other observed signals were proposed to be part of the same series of structures, bearing along with one Si=O zero, one (equal or similar to **11**), two (equal or similar to **12**), and five OH groups, or one OH and one CH_2_OH group (**13**). The products proposed to be **10**–**13** had survived heating to 220 °C for vaporization from aerosol particles before MS analysis (Avery et al. 2023).

The double bond equivalent (DBE) is a number that quantifies the hydrogen deficit mentioned above; it counts the number of double bonds plus rings in a molecular structure. For the molecular formula C_*c*_H_*h*_O_*o*_Si_*si *_it is defined as DBE = [2(*c* + *si*) + 2 − *h*]/2. For example, the parent compound D_5_ (C_10_H_30_O_5_Si_5_) has DBE = 1, corresponding to one ring and no double bond. Structures **10**–**13** exhibit one ring and one Si=O double bond each, in accordance with DBE = 2 that follows from the molecular formulas of observed MS signals. While the concept of double bond equivalents was discussed by Avery et al. (2023), proposed structures included only additional double bonds rather than additional rings.

Similarly, Chen et al. (2023) treated D_5_ in the gas phase with OH radicals, and by mass spectrometry they found in the resulting aerosol particles many siloxane products. Among several others, there were three MS signals with molecular formulas C_7_H_22_O_7_Si_5_, C_6_H_20_O_8_Si_5_, and C_7_H_22_O_8_Si_5_ that they hypothesized to correspond to possible structures **11**, **12**, and **13**, respectively, as above, each containing a Si=O functional group. The first two peaks were even dominant in the mass spectrum. Consideration of alternative structures without Si=O double bonds was not reported. The products in question were formed at 27–80% relative humidity in gas/aerosol particles that were collected on Teflon filters, were extracted therefrom with CH_3_CN under sonication, and concentrated under a gentle stream of N_2_ before MS analysis (Chen et al. 2023).

L_2_, D_4_, and D_5_ are typical pollutants in landfill gas and in biogas from anaerobic sludge digestion in wastewater treatment plants (CH_4_/CO_2_ mixtures). In order to develop a method to remove siloxanes from biogases for technical use, Divsalar et al. (2018) irradiated (λ = 185 and 254 nm) L_2_ or D_4_ in air or in artificial biogas at 50 °C and achieved decomposition into silica micro-particulates. During L_2_ irradiation, four byproducts were separated by GC (30–120 °C) that had molecular formulas C_8_H_24_O_4_Si_4_, C_4_H_12_O_3_Si_3_, C_4_H_12_O_3_Si_2_, and C_4_H_12_O_2_Si_2_ by MS. The authors stated the former to be D_4_, and the latter three to be **14**, **15**, and **16**, respectively. Irradiation of D_4_ yielded a byproduct C_6_H_18_O_5_Si_4_ stated to be **17**. No evidence beyond MS results was given for structures **14–17** that contain one or even two Si=O double bonds each (Divsalar et al. 2018).

Meepage et al. (2024) obtained from the reaction of D_5_ and OH radicals two instead of the expected one C_9_H_28_O_6_Si_5_ (by MS) compounds; these could be separated by liquid chromatography (UPLC) using aqueous salt solutions/acetonitrile mixtures as eluents. The authors detected these same two isomers also in the atmosphere of New York City. No structural information beyond MS^2^ was acquired. While one of these isomers is the often**-**found D_4_D^OH^, according to the authors the structure of the other is unknown, though “a plausible structure is a ring-opened product of D_4_D^OH^ with one unit of unsaturation (shown as the ring opened product of *m/z* 371 in Scheme 1)”, which latter is the anion ^–^O(Me_2_SiO)_4_Si(= O)Me.

We summarize the above facts as follows:

1. Structure proposals **1**–**17** containing a Si=O double bond are unprecedented, as none is the molecular structure of a known compound according to SciFinder (Chemical Abstracts Service, as of July 5, 2025).
2. There were no attempts to further elucidate the structures of the compounds in question (proposed structures **1–17**) beyond MS or MS/MS; that is, no macroscopic amounts were obtained, and no other spectra or properties were reported.
3. The compounds proposed by the respective authors to be **1**–**13** are described as stable; they formed typically at room temperature in a humid gas/aerosol and were found after workup procedures such as collecting aerosol particles on a filter, washing from filter material into CH_3_CN/H_2_O under sonication, followed by concentration. The compounds assigned by Divsalar et al. (2018) structures **14**–**17** survived gas chromatography at elevated temperatures, while Meepage et al.’s (2024) two C_9_H_28_O_6_Si_5_ isomers both survived liquid chromatography in aqueous eluents. In that sense, these are well-behaved compounds, which is very surprising in light of what is known about the reactivity of Si=O double bonds.

## Reactivity of compounds containing Si=O double bonds

Already in the early 1900 s, Kipping attempted to prepare silanones (R_2_Si=O) as silicon analogues of ketones, as well as other organic compounds with double bonds to Si atoms. All those attempts failed and instead led to oligo**-** or polymeric materials (Kipping 1912, 1937).

Important results of more than a century of silicon chemistry can be summarized as follows: Single bonds Si–O are very strong, far stronger than the π component of Si=O double bonds, so there is ample thermodynamic driving force to replace a Si=O by two Si–O bonds. In Table 2, energy values of Si–O single and double bonds are contrasted with those of C–O and Si–C single and double bonds, illustrating that silicon chemistry is vastly different from carbon chemistry. Note in particular that, in striking contrast to SiO chemistry, the π component of a C=O double bond is as strong or even stronger than its σ component or a C–O single bond. Ignoring this fundamental difference may lead to false analogies in silicon chemistry. Further, as expressed by the Pauling electronegativities of Si, C, and O (1.90, 2.55, and 3.44), a Si–O bond is strongly polarized in the sense Si^δ+^–O^δ–^ (much more so than a C–O bond); therefore, Si=O double bonds are extremely reactive, not viable under normal laboratory conditions, and therefore rare. In characteristic reactions, R–Si(= O)–R compounds add polar reagents (e.g., H_2_O) to gain two strong single bonds for a Si=O double bond, or undergo oligo- or polymerization to cyclic (SiR_2_–O–)_*n*_ or linear structures (…–SiR_2_–O–SiR_2_–O–…). According to theoretical studies, the cyclodimerization of silanones R_2_Si=O to cyclodisiloxanes (R_2_SiO)_2_ is exothermic by ~ 100 kcal/mol, and for R = H proceeds without barrier (Kudo and Nagase 1985; Kimura and Nagase 2001).

**Table 2:** Approximate energies of some bond types between Si, C, and O atoms

Bond	Energy (kcal/mol)	Reference
π component of Si=O	58.5	Suzuki et al. 1998
	64.2, 69.1	Avakyan et al. 2006
	58	Alvarado-Beltran et al. 2017
σ component of Si=O	119.7	Suzuki et al. 1998
	109	Alvarado-Beltran et al. 2017
Si–O single bond	114, 116, 128	Brook 2000
π component of C=O	95.3	Suzuki et al. 1998
	89	Mortimer and Müller 2020
σ component of C=O	93.6	Suzuki et al. 1998
C–O single bond	80	Mortimer and Müller 2020
π component of Si=C	31.6	Wang and Poirier 1998
	37–47	Brook 2000
	37.7, 39.6	Avakyan et al. 2006
Si–C single bond	88, 90	Brook 2000

For example, dimethylsilanone could be observed by IR in an argon matrix at 12 K, but oligomerized on warming to 40 K (Withnall and Andrews 1986; Khabashesku et al. 1986, 1988a), and was a short-lived intermediate in the vacuum thermal decomposition of PDMS (Kulyk et al. 2016). In much dedicated research, it became clear that silanones need stabilization of the Si=O double bond, either electronic or steric, to achieve a longer lifetime; for leading references, see Rodriguez et al. (2013), Ishida et al. (2015, 2023), Linden et al. (2015), Alvarado-Beltran et al. (2017), and Kobayashi et al. (2019, 2021). Stabilization of the tricoordinate Si atom in R_2_Si=O by a donor ligand leads to a tetracoordinate Si and severely disturbs the electron distribution, as do bonding of the Si atom to a transition metal complex (Filippou et al. 2014) or complexation of the O atom with an acceptor ligand. A stable (isolable at normal laboratory conditions under dry inert gas) silanone R–Si(= O)–R (R = alkyl) featuring a genuine (not electronically perturbed) Si=O bond was synthesized for the first time in 2019 (Kobayashi et al. 2019; for a review, see Loh and Aldridge 2021). This as yet unique compound owes its existence to a sterically extremely demanding di-alkyl substituent R–R, i.e., Ar_2_C–CH_2_CH_2_–CAr_2_ with Ar = 4-methoxy-3,5-di-*tert-*butylphenyl. Despite this exceptional steric protection, this silanone is highly reactive, electrophilic at the Si atom, and nucleophilic at the O atom. Its Si=O bond avidly adds several nucleophilic or electrophilic reagents. For example, a water molecule is added to form the corresponding geminal silanediol (R––R)Si(OH)_2_, and reagent B(C_6_F_5_)_3_ yields the corresponding adduct F_5_C_6_–Si(R––R)–O–B(C_6_F_5_)_2_. In the absence of such reagents, this silanone in hexane solution dimerizes immediately at room temperature to the corresponding highly congested cyclodisiloxane, a stable derivative of the elusive D_2_ (Kobayashi et al. 2019).

Si=O bonds bearing substituents other than alkyl, e.g., phenyl or alkoxy, are similarly reactive as alkylsilanones. Diphenylsilanone (Ph–Si(=O)–Ph) and dimethoxysilanone (MeO–Si(=O)–OMe) were observed by IR spectroscopy in an argon matrix at 12 K, but oligomerized to the corresponding cyclosiloxanes D^Ph2^_*n*_ and D^(OMe)2^_*n*_ (*n* = 2 or 3) on warming to 40 K (Khabashesku et al. 1988b, 1998). PhO–Si(=O)–Me was likewise obtained in an argon matrix below 30 K (Bornemann and Sander 2000).

While the SciFinder database for diethyl carbonate (EtO–C(=O)–OEt) lists about 35,000 references and 90 commercial suppliers (as of July 5, 2025), for the silicon analogue EtO–Si(=O)–OEt (RN 18954-71-7) there are no more than 20 references, none of which actually describes the synthesis or reports any experimental property of this compound. For RN 18954-71-7 two suppliers are listed, but without a structure or any experimental property or characterization given on the suppliers’ homepages, the identity of the material offered there remains dubious. All taken together, to the best of our knowledge, the literature does not provide any hint that simple substituents such as present in structures **1**–**17** (C, O, Si) could stabilize a Si=O bond.

As a summary, all studies known from the literature show that compounds with Si=O double bonds are not viable under ambient environmental or laboratory conditions unless stabilized by strong steric or electronic effects. However, even the stabilized compounds are highly reactive, e.g., towards water (Filippou et al. 2014; Kobayashi et al. 2019). For these reasons, it is extremely unlikely that compounds with Si=O double bonds form from simple D_*n*_ in ambient air and are stable in both the environment and the laboratory.

## Alternatives to Si=O double bonds in products formed from siloxanes and OH radicals under simulated environmental conditions

### Other double bonds

Since Si=O double bonds are not a realistic structural option for the stable “unsaturated” oxidation products of siloxanes, what structural alternatives are available? In the following discussion, we assume the molecular formulas reported were correctly derived from the mass spectra, and the presumed Si=O double bonds are not artifacts formed during analytical procedures, such as water elimination from silanediols. In fact, the mass spectrum of Me_2_Si(OH)_2_ (M = 92), even under 70 eV EI ionization, is dominated by *m*/*z* 77 (M–Me); there is no signal at *m*/*z* 74 (M–H_2_O) (Claflin et al. 2021; Dwivedi et al. 2013; compare also Wendler et al. (2001) and Kobayashi et al. (2019)).

Silenes (compounds carrying Si=C double bonds) are highly reactive as well; the π bond energy of Si=C is even lower than that of Si=O and far lower than that of the Si–C single bond (Table 2), resulting in silenes being stable only when strongly sterically protected by substituents on Si and C (Ottosson and Eklöf 2008; Baines 2013; Yang et al. 2020). The same is true for Si=Si double bonds (Hanusch et al. 2021).

Double bonds C=C or C=O are, in principle, an option, as in the case of silyl formates. Their formation requires a fundamental molecular reconstruction, since neither C–C nor C–O single bonds are present in methylsiloxanes. Silanols and silyl formates are formed by a shift of Si from C to O, strongly favored by thermodynamics (Table 2). Furthermore, the particular electron structure around a Si atom as a third row element facilitates Si shifts compared to C shifts, so Si shifts are far more common than C shifts, as shown by Kira and Iwamoto (2001).

For the molecular formulas considered here, mathematics provides strong evidence against C=C or C=O double bonds. Consider C_6_H_18_O_5_Si_4_, the molecular formula of the hypothetical D_3_D^=O^ (**17**). For this molecular formula, the mathematical structure generator MOLGEN 5.0 (Gugisch et al. 2014) is able to generate structures containing a C=C or a C=O double bond only if simultaneously at least a Si–H or Si–Si bond is allowed, both weak bonds and therefore improbable structural elements in a stable molecule. In contrast, bicyclic structures for this molecular formula are generated without the need for a Si–H or Si–Si bond, among them DTDT (**alt-17**, see below). Quite analogous results are obtained for C_8_H_24_O_6_Si_5_, the molecular formula of hypothetical D_4_D^=O^ or DTDDT, and for those corresponding to its hydroxyl derivatives **11**, **12**, and **13**. On the other hand, for the molecular formulas of known silyl formates D_3_D^OCHO^ (C_8_H_22_O_6_Si_4_) and D_4_D^OCHO^ (C_10_H_28_O_7_Si_5_), MOLGEN 5.0 generates structures containing a C=O double bond without any Si–H or Si–Si bonds, among them the said silyl formates. Hence, C=C or C=O double bonds are not the primary choice to explain DBE ≥ 2 in the stable “unsaturated” oxidation products under consideration here.

### Cycles as alternatives to double bonds

#### Known bicyclic and polycyclic siloxanes

The simplest structure of a possibly stable oxidation product of a D_*n*_ with DBE ≥ 2 is bicyclic, tricyclic, etc., containing no Si=O double bond. Bicyclic, tricyclic, and even polycyclic oligomeric methylsiloxanes are by no means exotic or unstable species; on the contrary, several such compounds have been known for decades and are well-characterized, as exemplified in Table 3.

**Table 3:** Structures and modes of formation of some bi-, tri- and polycyclic methylsiloxanes

Compound CAS-RN	Structure shorthand and type of skeleton	X-ray study or mode of formation: Reference
51717-82-9	T_2_D_3_ bicyclo[3.3.3]	X-ray: Menczel and Kiss (1975); Pyrolysis of a D/T silicone: Garzo and Alexander (1971); Pyrolysis of a branched chain polymer: Blazso et al. (1983);
17866-09-0	T_2_D_3_ bicyclo[5.3.1]	Pyrolysis of a T_2_D_6_ isomer: Makarova et al. (1983); Transannular cyclization of a *cis*-cyclopentasiloxane-2,6-diol: Timofeeva et al. (1984a)
51717-37-4	T_2_D_4_ bicyclo[7.3.1]	Pyrolysis of a T_2_D_6_ isomer: Makarova et al. (1983)
51717-38-5	T_2_D_4_ bicyclo[5.3.3]	X-ray: Menczel (1977); Pyrolysis of a T_2_D_6_ isomer: Makarova et al. (1983)
51717-36-3	T_2_D_4_ bicyclo[5.5.1]	X-ray: Polishchuk et al. (2001); Pyrolysis of a branched chain polymer: Blazso et al. (1983); Pyrolysis of a T_2_D_6_ isomer: Makarova et al. (1983); Transannular cyclization of a monocyclic *cis-* or *trans*−2,8-siloxanediol: Timofeeva et al. (1984a), Makarova et al. (1985), Chizhova et al. (2004)
87122-51-8	T_4_D_3 _tricyclo[7.5.1.1^5,11^]	Pyrolysis of a branched chain polymer: Blazso et al. (1983)
17865-85-9	[MeSiO_3/2_]_8_ cube-shaped (T_8_), DBE = 5	X-ray: Larsson (1960); Hydrolysis/condensation of MeSiCl_3_: Olsson (1959); Hydrolysis/condensation of D^OEt^_4_: Handke et al. (2008)
18106-15-5	[MeSiO_3/2_]_10_ pentaprism-shaped (T_10_), DBE = 6	X-Ray: Baidina et al. (1980); Hydrolysis/condensation of MeSiX_3_: Larsen et al. (2022)

Table 3 shows

- Bicyclic methylpenta- and hexasiloxanes are stable even at high temperature, as some were formed by pyrolysis reactions,
- Bicyclic methylsiloxanes form spontaneously by intramolecular (transannular) cyclization (Makarova et al. 1985; Wachholz et al. 1995),
- Even penta- and hexacyclic methylsiloxanes (POSS silsesquioxanes) form surprisingly easily by hydrolysis/condensation of MeSiX_3_, so that T_8_ and T_10_ are well-known compounds (Hartmann-Thompson 2011; Kowalewska 2017; Larsen et al. 2022). Derivatives of T_8_ are even applied in several industrial sectors (Olejnik et al. 2022; Bialek and Czaja 2023).

Some further bi-, tri-, and polycyclic methyl- and phenylsiloxanes with structures proven by single-crystal X-ray analysis and formed by intramolecular condensation are shown in Table S1 in the Supporting Information. Among these, there are even molecules as small as a bicyclo[3.3.1]tetrasiloxane (an O-bridged D_4_ derivative, CAS-RN 639469-71-9) and a tricyclo[5.3.1.1^3,9^]pentasiloxanol (a doubly O-bridged D_5_ derivative, as such containing a bicyclo[3.3.1]tetrasiloxane substructure, CAS-RN 639469-69-5, Unno et al. 2003).

The examples in Tables 3 and S1 show that strain in these ring systems is not prohibitive. In fact, ring strain in D_3_, D_4_, and D_5_ was reported to be merely 2.5, 0.24, and 0.25 kcal/mol, respectively (Voronkov 1996). Highly substituted derivatives of D_2_ such as D^Mes2^_2_ (Mes = mesityl, Fink et al. 1984), D^*t*Bu2^_2_ (Qing and Cui 2017), or D^R–R^_2_ (R–R as above, Kobayashi et al. 2019) are stable compounds demonstrating that even this smallest cyclic siloxane system is not energetically unattainable. The spontaneous dimerization of Kobayashi’s silanone is obviously exothermic despite the product D^R–R^_2_ being highly congested.

#### A possible reaction forming bi- or tricyclic siloxanes

A possible reaction for the intramolecular (transannular) formation of bi- or tricyclic siloxanes is silanol condensation. This reaction type is used on an industrial scale (mostly intermolecularly) and is the basis of the silicone industry at large. It is a nucleophilic substitution, with a Si–OH oxygen atom acting as the nucleophile at a second Si atom bearing a leaving group; four widely used variants are the following:

- hydrogen chloride as leaving group, *Si*–OH + Cl–*Si′* → *Si*–O–*Si′* + HCl,
- water as leaving group, *Si*–OH + HO–*Si′* → *Si*–O–*Si′* + H_2_O,
- alcohol as leaving group, *Si*–OH + RO–*Si′* → *Si*–O–*Si′* + ROH,
- carboxylic acid as leaving group, *Si*–OH + R–CO–O–*Si′* → *Si*–O–*Si′* + RCOOH.

Variants a and b are fundamental reactions in siloxane chemistry. They are often used inter- and intramolecularly in preparative siloxane chemistry, mainly to produce siloxane chains and networks (Sarich et al. 1990; Sugiyama et al. 2019; several examples in Tables 3 and S1). Reactions c, in particular with R = CH_3_ or C_2_H_5_ (alcohol curing), and d with R = CH_3_ (acetate curing) are widely used in silicone crosslinking, e.g., in the curing of silicone sealants. Formic acid as a leaving group (reaction d with R = H) would be a logical extension of this chemistry. An O-bridged bicyclic product of a D_*n*_ may, thus, form from a cyclosiloxanediol (reaction variant b) or from an intermediary cyclosiloxanol-silyl formate (variant d, R = H). Alton and Browne (2022) observed in the OH radical oxidation of D_3_ that of all products identified and quantified in the gas phase, the bifunctional one (silanol and formate ester) was the only one suffering significant loss as soon as no further oxidant was produced in the reaction chamber. This observation is compatible with the occurrence of such a cyclization, though other explanations are of course possible, e.g., adsorption to the chamber walls.

While this reaction type produces bridges consisting of a single O atom, bridges made of a single CH_2_ group are also known (Delman et al. 1969; Zamaev et al. 1988) and were observed during OH radical oxidation of D_*n*_ (Wu and Johnston 2016). As far as we know, the mode of formation of the latter is unknown, but their formation is not implausible under radical conditions.

Generally, transannular cyclizations seem to be favored in substituted cyclosiloxanes compared to substituted cycloalkanes by the formers’ increased molecular flexibility (Timofeeva et al. 1984b), which is due to the low rotational barrier about the Me_2_Si–O bond (0.6 kcal/mol, Cypryk 2007) and the highly flexible Si–O–Si bond angle (86° to 180° observed in X-ray studies, Fink et al. 1984; linearization barrier 0.3 kcal/mol, Cypryk 2007). Moreover, silanols are “sticky”, prone to (self-)condensation, while alcohols are not; compare, as a simple example, the behavior of trimethylsilanol (Cella and Carpenter 1994) to that of *tert*-butanol.

**Fig. 2:**
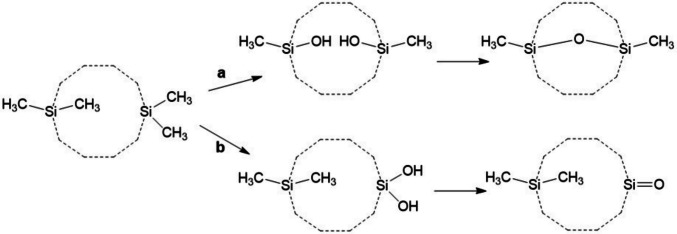
The molecular formula of a structure containing a Si=O double bond is identical to that of a structure bearing an additional Si–O–Si bridge instead of the Si=O bond

In a methylsiloxane molecule, two CH_3_ groups on different Si atoms may independently be oxidized by OH and replaced to form two *Si*–OH groups by the often observed, though mechanistically not completely understood, reaction sequence (Fig. 2, path a). An intramolecular condensation may then occur under the loss of a water molecule. The resulting cyclic siloxane is isomeric to the product of a hypothetical sequence of two oxidation steps in one and the same Si(CH_3_)_2_ group (→ Si(OH)_2_, Fig. 2, path b) followed by the loss of a water molecule to produce a Si=O double bond. Thus, for a hypothetical siloxane structure containing a Si=O double bond, in most cases several alternative isomeric structures are possible that exhibit an additional ring (an O-bridge) instead of the Si=O double bond.

Our hypothesis is that in the experiments considered here, cyclosiloxane-1,*n*-diols can intramolecularly condense, forming a bridge and thus bicyclosiloxanes (Fig. 2, path a). There is ample precedent of silanol condensations occurring intra- or intermolecularly. Usually, intermolecular silanol condensations are undesired or intended secondary reactions in the hydrolysis in solution of chlorosilanes, alkoxysilanes, or siloxysilanes to silanols in the presence of acidic or basic catalysts. There are also hints of intermolecular silanol condensations occurring in the absence of catalysts, simply by warming to 100 °C in solution (Kantor 1953), without warming in a smog chamber (“dimers” found by Sommerlade et al. 1993; Wu and Johnston 2016, 2017; Lewine et al. 2025) or to intramolecular silanol condensation simply on storage (Makarova et al. 1985). In a gas phase (in the atmosphere or in a reaction chamber), catalysis is not expected, nor was heating provided in the experiments cited. The silanol condensations proposed here are all intramolecular, and generally, intramolecular reactions are enormously accelerated as compared to intermolecular ones, due to reaction centers being close to one another, particularly in small rigid molecules. Therefore, intramolecular silanol condensations may not require catalysis and may reasonably occur in the gas phase. However, if in the experiments considered here the compounds under discussion were formed in a condensed phase (adsorbed to the chamber walls or in aerosol particles), catalysis may play some role.

#### Cyclic siloxane structures without Si=O as alternatives for structures 1–17

Of course, two Si atoms to be joined by an oxidation/condensation sequence are not necessarily located in a common ring or in a ring at all. For structures **1–9**, still assuming a cyclopentasiloxane bearing a linear trisiloxane, and excluding strained cyclodisiloxanes, not only bicyclo[5.3.1] systems may be formed (involvement of two Si atoms in the cyclopentasiloxane ring) but also:

- bicyclo[5.3.3] or bicyclo[7.3.1] systems (a ring Si atom and the first side chain Si atom),
- bicyclo[5.5.3] or bicyclo[7.5.1] systems (a ring Si atom and the second side chain Si atom),
- bicyclo[7.5.3] or bicyclo[7.7.1] systems (a ring Si atom and the third side chain Si atom),
- spiro[5.9] or spiro[7.9] systems (the Si atom bearing the trisiloxane side chain and the second or third side chain Si atom),
- a cyclopentasiloxane connected by an O atom to a cyclotrisiloxane (the first and the third side chain Si atoms).

**Fig. 3:**
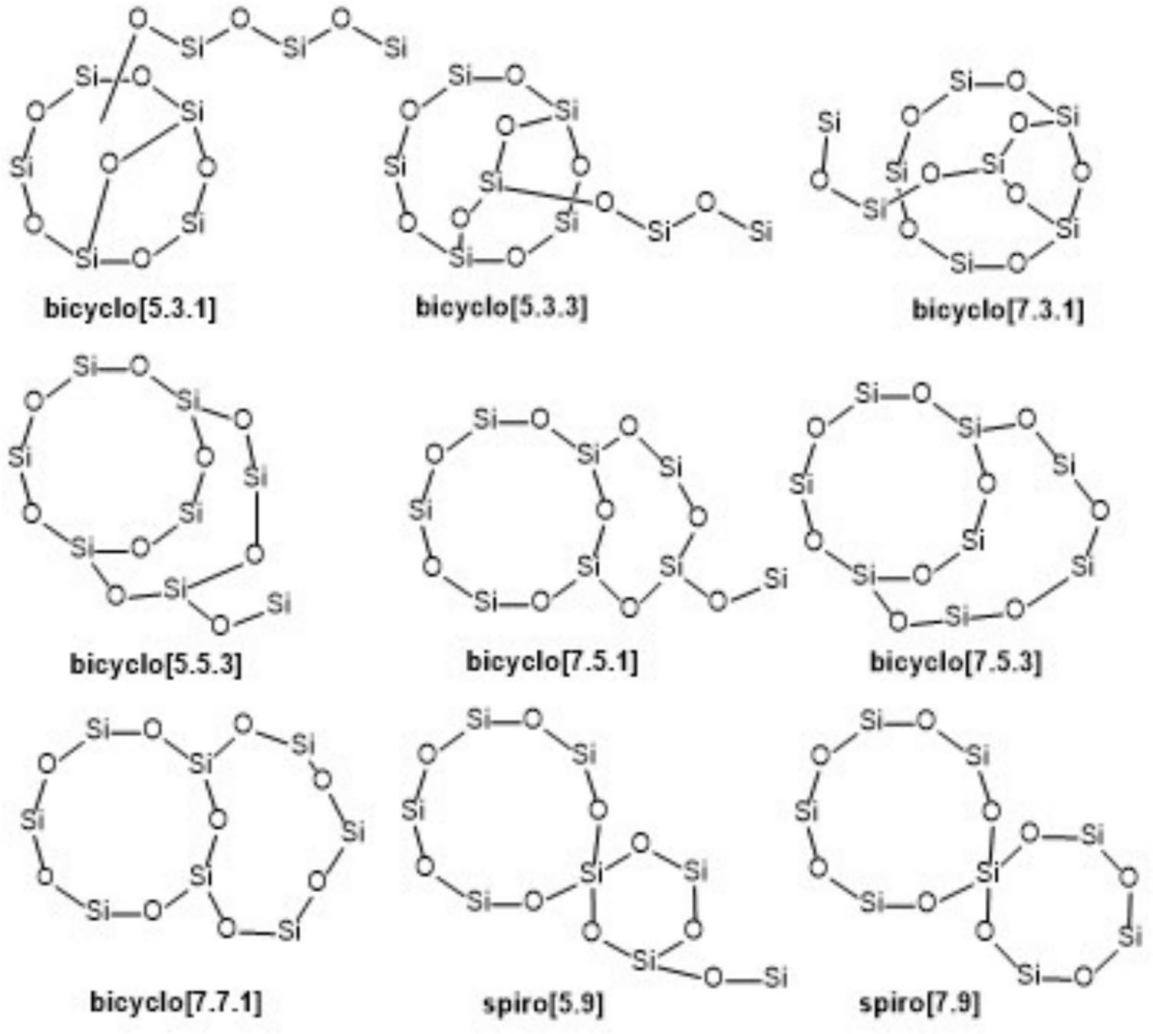
Possible alternative structures for **1**–**9** that may form by oxidation/condensation from compounds based on the cyclopentasiloxane plus trisiloxane skeleton. For clarity, only the siloxane backbone is shown. In each structure, the 14 free sites are occupied by 10 CH_3_ and 4 OH groups for alternatives to **1**, or by 11 CH_3_ and 3 OH groups for alternatives to **2**–**9**

For most of these structures, see Fig. 3. All these bicyclic siloxane systems are known; some examples are given in Tables 3 and S1.

**Fig. 4:**
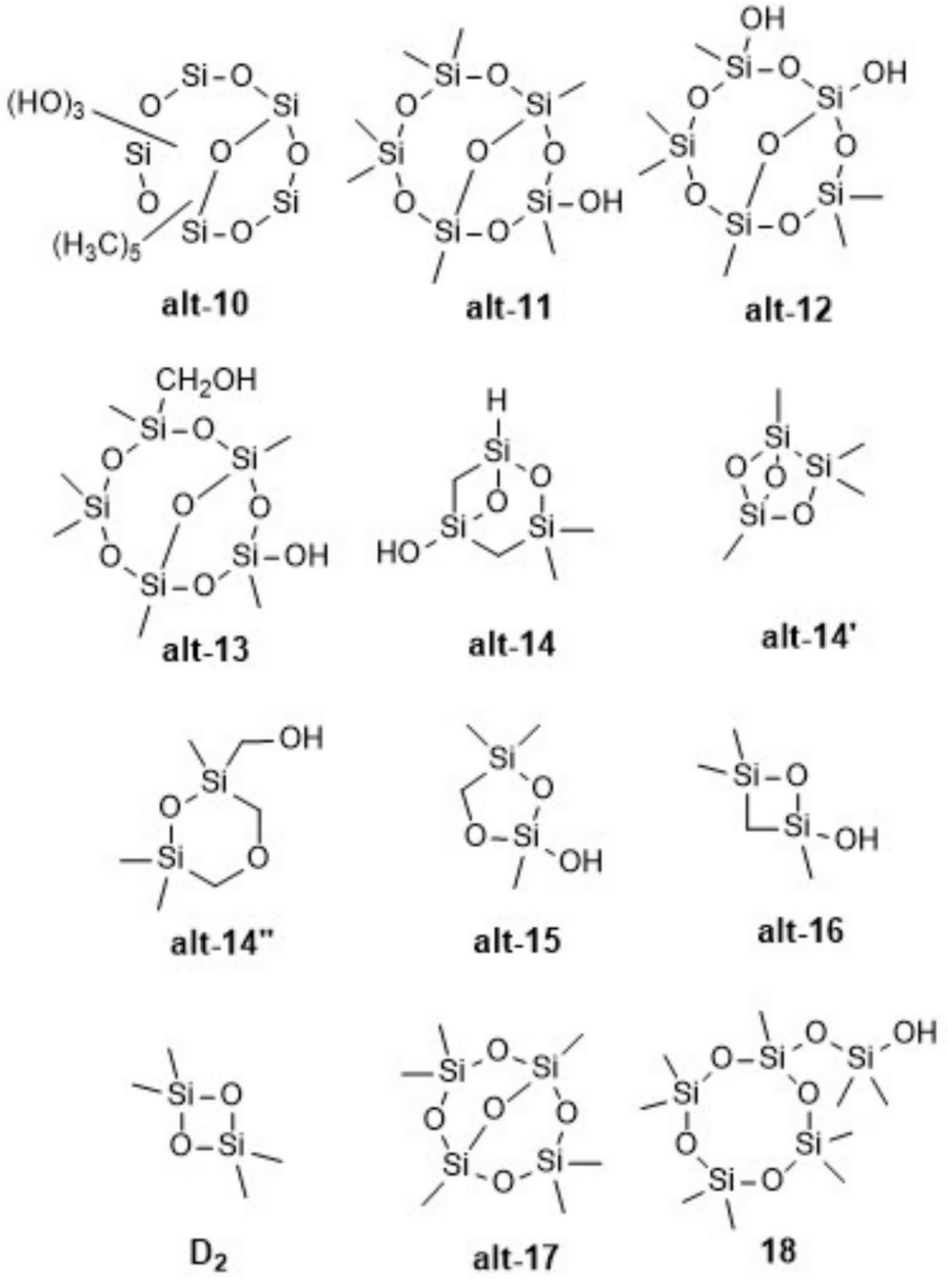
Possible alternative structures for **10**–**17** and for the second C_9_H_28_O_6_Si_5_ isomer

Therefore, as more plausible alternatives for structures **10–13**, the corresponding bicyclo[5.3.1]pentasiloxane structures **alt-10** to **alt-13** should be considered (Fig. 4); again, location of functional groups within the molecules is not determined.

For structures **14**–**17**, we constructed alternatives, supported by the structure generator MOLGEN 5.0 (Gugisch et al. 2014). We do not speculate on possible ways of formation of alternatives to **14**–**16** since they were formed under high-energy conditions (185 nm irradiation, equivalent to 154.7 kcal/mol), where the irradiation is able to cleave all kinds of bonds resulting in a bunch of highly reactive and unselective radicals (Divsalar et al. 2018). In the following numbers of isomers, stereoisomerism is neglected.

Under the restrictions that bond types O–O, Si–Si, Si–H (weak bonds potentially highly reactive under the reaction conditions of Divsalar et al. (2018)) and double bonds are excluded, the software generates 43 monocyclic alternatives for **15** (C_4_H_12_O_3_Si_2_, DBE = 1), among them **alt-15** (Fig. 4). Under the same restrictions, for **16** (C_4_H_12_O_2_Si_2_, DBE = 1), four alternatives are generated, among them **alt-16** and D_2_. The molecular formula of **14** (C_4_H_12_O_3_Si_3_, M = 192, DBE = 2) poses difficulties due to a high DBE in a very small molecule and a high number of Si atoms. Under the full set of restrictions, MOLGEN 5.0 is unable to generate a structure for this molecular formula. Structures are found only if restrictions are partially released. E.g., if one Si–H bond is allowed, 99 structures are generated, among them the one given here as **alt-14**. If one Si–Si bond is allowed but no Si–H bond, then **alt-14′** is one among 80 structures generated. These two structures are not convincing due to the Si–H and the Si–Si bond. However, since Divsalar et al. (2018) did not report details on how they assigned molecular formulas to MS signals of volatile byproducts (“GC–MS”), even the molecular formula of the compound considered as **14** may be erroneous. For a possible alternative molecular formula of the same integer molecular mass, C_6_H_16_O_3_Si_2_ (M = 192, DBE = 1), MOLGEN 5.0 generates, under the full set of restrictions, 2162 structures without Si=O, Si–H, or Si–Si bonds, among them the one given here as **alt-14″**.

According to what was demonstrated above, an obvious alternative for monocyclic **17** is the bicyclo[3.3.1]tetrasiloxane **alt-17**. The structures of two substituted bicyclo[3.3.1]tetrasiloxanes comprising an O-bridge were previously verified by X-ray analysis (CAS-RN 639469-71-9 and 639469-69-5, Table S1, Unno et al. 2003).

For the unexpected second C_9_H_28_O_6_Si_5_ isomer (Meepage et al. 2024), we propose to consider structure D_3_D^OSiMe2OH^ (**18**). This compound may be formed from D_4_D^OH^ by intramolecular nucleophilic substitution (OH or O^–^ group attack at a non-vicinal Si atom), or from some reactive intermediate on the way to D_4_D^OH^. This potential reaction may be accelerated by the closeness of attacking and attacked atoms in the intramolecular reaction, compensating for the lack of a typical leaving group. Both the ring contraction in a cyclopentasiloxane and a silanol or silanolate group acting as a leaving group therein are not without precedent (Varaprath 1999) and are in line with ring strain in D_4_ not being higher than in D_5_, while the alternative structure D_2_D^OSiMe2OSiMe2OH^ seems less likely due to ring strain in a cyclotrisiloxane (Voronkov 1996). The striking similarity of the product ion mass spectra of Meepage et al.’s (2024) two isomers also has precedent in the mass spectra of D_5_ and D_3_D^OSiMe3^ (Varaprath 1999).

#### Bicyclic and tricyclic structures without Si=O as alternatives for other “unsaturated” oxidation products of D_5_

**Fig. 5:**
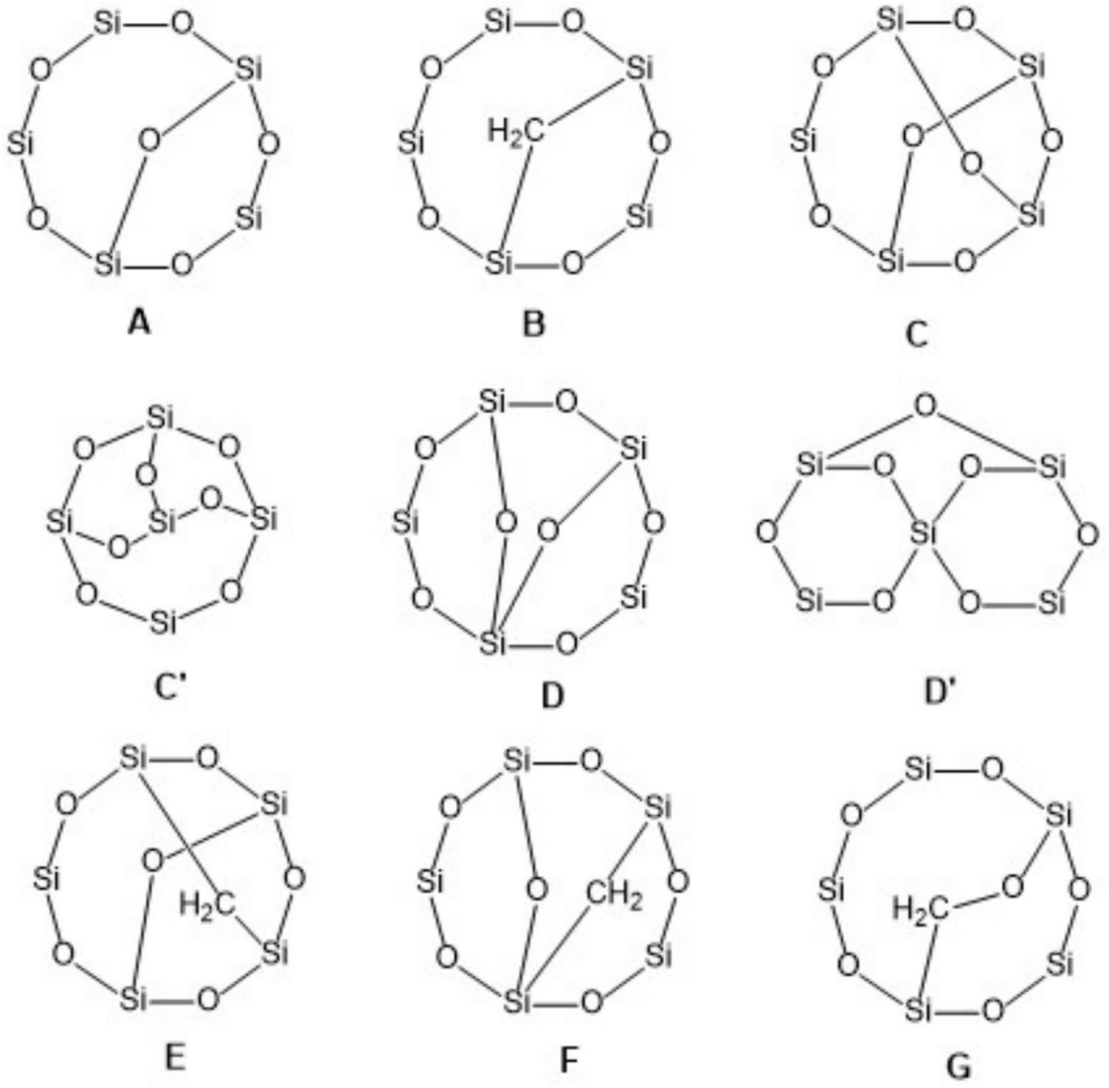
Bi- and tricyclic siloxane backbones underlying the potential structures in Table 4. In each ring system, the free sites (eight in bicyclics, six in tricyclics) are occupied as detailed in Table 4

For all Si_5_ products of DBE = 2 or 3 from the OH radical oxidation of D_5_ published as molecular formulas without structures by Wu and Johnston (2017) and by Chen et al. (2023), we generated possible structures without Si=O double bonds (in fact without any double bonds), still containing the cyclopentasiloxane substructure. Structures constructed for all these molecular formulas are shown in Table 4/Fig. 5. Structures considered here are obtained from D_5_ by minimal structural changes, that is, they still contain a cyclopentasiloxane substructure, as emphasized in drawings **A–G** and less easily perceived in alternative equivalent drawings, e.g., **C′** or **D′**. In contrast, there are lots of isomeric possible structures no longer containing a cyclopentasiloxane substructure that, if present in the D_5_ product mixture, would require more fundamental rearrangements. The tricyclic ring system **C** (tricyclo[5.3.1.1^3,9^]) is realized in compound CAS-RN 639469-69-5 (see the Supplementary Information, Table S1, Unno et al. 2003), whereas tricyclic **D** (tricyclo[5.3.1.1^1,5^]) appears highly strained and its realization as a compound is questionable. **D** is a substructure in tetracyclic CAS-RN 2060413-97-8, a dubious structure in itself.

**Table 4:** Possible structures without Si=O double bonds for Si_5_ molecular formulas of DBE = 2 or 3 that appear in the papers of Wu and Johnston (2017) and Chen et al. (2023) as oxidation products of D_5_. For ring systems **A–G**, see Fig. 5

Molecular formula	DBE	Occurrence in Wu and Johnston (2017)^a^	Occurrence in Chen et al. (2023)^b^	Possible structures (exemplary): Ring system, substituents
C_8_H_24_O_6_Si_5_	2	Line 25	Table S2, Line 24	**A**, 8 CH_3_ or **B**, 7 CH_3_, 1 OH
C_7_H_22_O_7_Si_5_	2	Line 30	Table S3, Line 9	**A**, 7 CH_3_, 1 OH (**alt-11**) or **B**, 6 CH_3_, 2 OH
C_8_H_24_O_7_Si_5_	2	Line 31		**A**, 7 CH_3_, 1 CH_2_OH or **B**, 6 CH_3_, 1 OH, 1 CH_2_OH or **G**, 7 CH_3_, 1 OH
C_6_H_20_O_8_Si_5_	2	Line 35	Table S3, Line 10	**A**, 6 CH_3_, 2 OH (**alt-12**)
C_7_H_22_O_8_Si_5_	2	Line 36	Table S3, Line 11	**A**, 6 CH_3_, 1 OH, 1 CH_2_OH (**alt-13**) or **G**, 6 CH_3_, 2 OH
C_5_H_18_O_9_Si_5_	2	Line 39		**A**, 5 CH_3_, 3 OH (**alt-10**)
C_7_H_22_O_10_Si_5_	2	Line 44		**A**, 4 CH_3_, 1 OH, 3 CH_2_OH
C_9_H_26_O_5_Si_5_	2	Line 23	Table S2, Line 23	**B**, 8 CH_3_
C_9_H_26_O_6_Si_5_	2	Line 26		**B**, 7 CH_3_, 1 CH_2_OH or **G**, 8 CH_3_
C_6_H_18_O_7_Si_5_	3	Line 29	Table S2, Line 22	**C**, 6 CH_3_ or **D**, 6 CH_3_
C_5_H_16_O_8_Si_5_	3	Line 34	Table S3, Line 8	**C**, 5 CH_3_, 1 OH or **D**, 5 CH_3_, 1 OH
C_7_H_20_O_6_Si_5_	3	Line 24	Table S2, Line 21	**E**, 6 CH_3_ or **F**, 6 CH_3_

^a^In Table S4 in the Supporting Information of Wu and Johnston (2017)

^b^In Tables S2 or S3 in the Supplementary Information of Chen et al. (2023)

A−CH_2_− bridge in a small cyclic siloxane similar to **B**,** E**, and **F** is realized in compound CAS-RN 119934-06-4, while bridges of length 2 (−CH_2_CH_2_−) similar to that in **G** (−CH_2_O−) are realized in compounds CAS-RN 119352-04-4 and CAS-RN 22588-76-7 (Supplementary Information, Table S1).

## Conclusion

Structures containing Si=O double bonds were recently proposed for some isolable products of OH radical-induced oxidation of cyclic dimethylsiloxanes under (simulated) environmental conditions. These structures were based prematurely on molecular formulas resulting from mass spectrometric techniques. We in the present work show that the experimental results may be better interpreted in terms of cyclic structures, as all experience shows that Si=O double bonds (unless strongly protected by substituents or at very low temperature) are extremely reactive and do not survive usual laboratory procedures. We demonstrate that the molecular formulas in question can easily be explained by structures comprising an additional ring instead of a Si=O double bond, and that many such bi-, tri-, or polycyclic siloxane ring systems are realized in stable, well-characterized compounds, some of which are even used in industry. For the case of the additional ring being an oxygen bridge between Si atoms (Si–O–Si), we show that known siloxane reactions (OH radical-induced replacement of CH_3_ by OH groups followed by intramolecular silanol condensation) suffice to explain the bridge formation. Overall, no evidence seems to exist for the occurrence of Si=O double bonds in the products of OH radical-induced oxidation of dimethylsiloxanes in the atmosphere.

## Supplementary information

Below is the link to the electronic supplementary material.

ESM 1 (DOCX 537 KB)

## Data Availability

Data will be made available upon reasonable request.
