# Supplementary material for: Atmospheric oxidation of dimethylsiloxanes, a source of Si=O double bonds?
Source: Environ Sci Pollut Res Int. 2025 Nov 14;32(46):26611–28. doi: 10.1007/s11356-025-37108-6 (PMC12672758; doi:10.1007/s11356-025-37108-6)
Supplement: Supplementary file 1 — (DOCX 537 KB) [file 11356_2025_37108_MOESM1_ESM.pdf]

Supplementary Information to

Atmospheric oxidation of dimethylsiloxanes, a source of Si=O double bonds?

Christoph Rücker*[1], Dennis Troegel[2], Klaus Kümmerer[1]

[1] Institute for Sustainable Chemistry, Leuphana University Lüneburg, Universitätsallee 1, D-21335 Lüneburg, Germany

[2] Department of Applied Chemistry, Nuremberg Institute of Technology, Kesslerplatz 12, D-90489 Nuremberg, Germany

Table S1. Some bi-, tri- and polycyclic methyl- and phenylsiloxanes with X-ray structure available, that were formed by inter- and intramolecular silanol condensation

**Compound CAS-RN**	**Structure shorthand and type of skeleton**	**Compound name [a]**	**Reference**
49538-51-4<br>73395-20-7	$T^{Ph}_2D_4$<br>bicyclo[5.5.1]	3,3,5,5,9,9,11,11-Octamethyl-1,7-diphenyl-bicyclo[5.5.1]hexasiloxane	Wu and Launer 1973; Shklover et al. 1981a; Unno et al. 1999
60096-10-8	$T^{Ph}_4D_3$<br>tricyclo-[7.5.1.1^{3,7}]	5,5,11,11,13,13-Hexamethyl-1,3,7,9-tetraphenyltricyclo[7.5.1.1^{3,7}]heptasiloxane	Shklover et al. 1981a
38049-91-1	$T^{Ph}_4D_2$<br>tricyclo-[7.3.1.1^{3,7}]	*trans*-5,5,11,11-Tetramethyl-1,3,7,9-tetraphenyltricyclo[7.3.1.1^{3,7}]hexasiloxane	Shklover et al. 1981b
3511-12-4	$T^{Ph}_4D_2$<br>tricyclo-[7.3.1.1^{3,7}]	*cis*-5,5,11,11-Tetramethyl-1,3,7,9-tetraphenyl-tricyclo[7.3.1.1^{3,7}]hexasiloxane	Shklover et al. 1979
3511-13-5	$T^{Ph}_4D_4$<br>tricyclo-[9.5.1.1^{3,9}]	*cis*-5,5,7,7,13,13,15,15-Octamethyl-1,3,9,11-tetraphenyltricyclo[9.5.1.1^{3,9}]octasiloxane	Shklover et al. 1978a
5256-79-1	T^{Ph}_8	Octaphenyloctasilsesquioxane	Shklover et al. 1978b
639469-71-9	$T^{TIP}_2(D^{TIP,OH})_2$ [b]<br>bicyclo[3.3.1]	1,3,5,7-Tetrakis(2,4,6-triisopropylphenyl)-bicyclo[3.3.1]tetrasiloxane-3,7-diol	Unno et al. 2003a

639469-69-5	tricyclo-[5.3.1.1^{3,9}]	1,3,5,7,9-Pentakis(2,4,6-triisopropylphenyl)-tricyclo[5.3.1.1^{3,9}]pentasiloxan-5-ol	Unno et al. 2003a
849727-69-1	$(T^{Tip})_6$ [b] tetracyclo-[5.5.1.1^{3,11}.1^{5,9}]	1,3,5,7,9,11-Hexakis(2,4,6-triisopropylphen-yl)tetracyclo[5.5.1.1^{3,11}.1^{5,9}]hexasiloxane	Unno et al. 2003b
119352-04-4	bicyclo[5.5.2]	1,3,3,5,5,7,9,9,11,11-Decamethyl-bicyclo[5.5.2]hexasiloxane	Ovchinnikov et al. 1989
22588-76-7	bicyclo[3.3.2]	1,5-Dimethyl-3,3,7,7-tetraphenyl-bicyclo[3.3.2]tetrasiloxane	Ovchinnikov et al. 1986
119934-06-4	bicyclo[3.3.1]	1,5-Dimethyl-3,3,7,7-tetraphenyl-bicyclo[3.3.1]tetrasiloxane	Zamaev et al. 1988

[a] Names are taken from SciFinder, [b] Tip = 2,4,6-triisopropylphenyl

Ovchinnikov YE, Shklover VE, Struchkov YT, Astapova TV, Zamaev IA, Zhdanov AA (1989) 1,3,3,5,5,7,9,9,11,11-Decamethyl-2,4,6,8,10,12-hexaoxa-1,3,5,7,9,11-hexasilabicyclo[5.5.2]tetradecane. Acta Cryst C 45:71-73.

Ovchinnikov YE, Shklover VE, Struchkov YT, Astapova TV, Zhdanov AA (1986) Structures of two cyclotetrasiloxanes with intra and intercyclic –CH_2CH_2–bridges. Z Anorg Allg Chem 533:159-164.

Shklover VE, Chekhlov AN, Struchkov YT, Makarova NN, Andrianov KA (1978a) The crystal structure of cyclic siloxanes and silazanes. IX. cis-1,1,7,7,9,9,15,15-Octamethyl-3,5,11,13-tetraphenyltricyclodecasiloxane. J Struct Chem 19:929-943.

Shklover VE, Struchkov YT, Makarova NN, Andrianov KA (1978b) Crystal structure of cyclic siloxanes and silazanes. X. Octa(phenylsilasesquioxane). J Struct Chem 19:944-954.

Shklover VE, Struchkov YT, Klement'ev IY, Tikhonov VS, Andrianov KA (1979) Crystal structure of cyclic siloxanes and silazanes. XIII. 1,1,7,7-Tetramethyl-3,5,9,11-tetraphenyltricyclohexasiloxane, isomer with melting point 162.5°C. J Struct Chem 20:251-256.

Shklover VE, Struchkov YT, Makarova NN, Zhdanov AA (1981a) Crystal structure of organosilicon compounds. XXVI. cis-1,7-Diphenyl-3,3,5,5,9,9,11,11-octamethylbicycloheptasiloxane and cis-1,1,7,7,9,9-hexamethyl-3,5,11,13-tetraphenyltricyclononasiloxane. J Struct Chem 22:561-569.

Shklover VE, Klement'ev IY, Struchkov YT (1981b) Crystal structure of siloxanes and silazanes. XXIV. 1,1,7,7-Tetramethyl-3,5,9,11-tetraphenyltricyclohexasiloxane, isomer with melting point 222.5°. J Struct Chem 22:372-376.

Unno M, Alias SB, Arai M, Takada K, Tanaka R, Matsumoto H (1999) Synthesis and characterization of cage and bicyclic silsesquioxanes via dehydration of silanols. Appl Organomet Chem 13:303-310.

Unno M, Tanaka T, Matsumoto H (2003a) Tip-substituted cage and cyclic silanols. J Organomet Chem 686:175-182.

Unno M, Imai Y, Matsumoto H (2003b) Hexakis(2,4,6-triisopropylphenylsilsesquioxane). Silicon Chem 2:175-178.

Wu TC, Launer PJ (1973) Some new cyclic organopolysiloxanes. J Chem Eng Data 18:350-352.

Zamaev IA, Ovchinnikov YE, Shklover VE, Struchkov YT, Astapova TV, Zhdanov AA (1988) Crystal structure of organosilicon compounds. Part LVII. 1,5-Dimethyl-3,3,7,7-tetraphenylbicyclo[3.1.3]-1,3,5,7-tetrasila-2,4,6,8-tetraoxanonane. Organomet Chem USSR 1:688-691.
